# Cyclopiazonic Acid Induces Mitochondrial Oxidative Stress in SH-SY5Y Cells: Protective Effects of Extra Virgin Olive Oil Phenolics

**DOI:** 10.3390/toxins18060252

**Published:** 2026-06-02

**Authors:** Carmen Martínez-Alonso, Yelko Rodríguez-Carrasco, María-José Ruiz

**Affiliations:** Research Group in Alternative Methods for Determining Toxics Effects and Risk Assessment of Contaminants and Mixtures (RiskTox), Laboratory of Food Chemistry and Toxicology, Faculty of Pharmacy and Food Science, University of Valencia, 46100 Burjassot, Spain; carmen.martinez-alonso@uv.es (C.M.-A.); m.jose.ruiz@uv.es (M.-J.R.)

**Keywords:** cyclopiazonic acid, SH-SY5Y cells, mitochondrial oxidative stress, neurotoxicity, EVOO phenolics, nrf2–keap1

## Abstract

Cyclopiazonic acid (CPA), a neurotoxin produced by Penicillium and Aspergillus genera, induces oxidative stress and neuronal damage, mechanisms implicated in neurodegenerative diseases. This study investigates the oxidative stress induced by CPA in SH-SY5Y human neuroblastoma cells, focusing on mitochondrial membrane potential, mitochondrial superoxide levels, ROS production, lipid peroxidation and gene expression. Additionally, the cytoprotective effects of extra virgin olive oil (EVOO) extract, along with its major polyphenols oleuropein (OLE) and tyrosol (TYR), were evaluated. CPA exposure increased mitochondrial superoxide levels and lipid peroxidation, reducing mitochondrial membrane potential, although no intracellular ROS generation was observed. Gene expression analysis revealed downregulation of antioxidant defense genes (nrf2, nos2, ho1, cat, keap1, nqo1, gpx1 and gsr), with the strongest repression observed for nos2 (93%), nqo1 (83%) and ho1 (79%) at the highest CPA concentration, consistent with oxidative stress markers. EVOO extract demonstrated protective effects, enhancing cell viability across all CPA assayed concentrations (400–600 nM). Conversely, TYR and OLE exhibited variable and concentration-dependent effects, also showing protection to a lesser extent, while EVOO extract proved to be more effective due to synergistic interactions among its phenolic components. Overall, CPA induces mitochondrial oxidative damage as a key mechanism of neurotoxicity, while EVOO phenolics mitigate this toxicity.

## 1. Introduction

Oxidative stress plays an essential role in the pathophysiology of various neurodegenerative diseases (NDs), including Alzheimer’s disease, motor neuron disease and Parkinson’s disease. It is defined as an imbalance between the production of reactive oxygen species (ROS) and cellular antioxidant defense mechanisms, leading to oxidative damage to macromolecules such as lipids, proteins and DNA. This damage leads to cellular dysfunction and disruption of homeostasis, and it ultimately culminates in cell death [1]. Neuronal cells are particularly vulnerable to oxidative damage due to their high metabolic rate, lipid-rich membranes in polyunsaturated fatty acids and weak antioxidant defenses, making them especially susceptible to oxidative stress, which is closely linked to neurotoxicity and disease progression [2,3]. The SH-SY5Y cell line, obtained from a bone marrow biopsy of metastatic neuroblastoma tissue, has become a widely used in vitro model for studying the complex pathogenesis of NDs, offering insights into disease mechanisms, identifying potential therapeutic aims, and aiding in drug discovery, due to its ease of use, reproducibility and scalability [4].

Cyclopiazonic acid (CPA) is a neurotoxic compound belonging to the indole-hydrindane-tetramic acid alkaloids family, structurally related to ergot toxins. It is produced by specific fungal species from the Penicillium and Aspergillus genera and has raised significant concerns due to its toxicological effects on both human and animal health. CPA has been detected in various food products, including cheeses, figs, maize, rice, peanuts, millet, poultry meat and animal feed, posing a potential threat to food safety [5,6]. The toxic effects of CPA often include symptoms such as weight loss, diarrhea, muscle and visceral degeneration, necrosis, convulsions, and, in severe cases, death [7]. These effects are primarily linked to CPA’s mechanism of action, which involves the inhibition of calcium-dependent ATPases and the subsequent disruption of intracellular calcium homeostasis [8]. Additionally, CPA induces oxidative stress, leading to inflammation in target tissues and potential pathological abnormalities [9].

Despite these findings, there remains a significant gap in research exploring strategies to mitigate CPA-induced oxidative stress. Developing neuroprotective agents or interventions to mitigate oxidative damage could provide valuable insights into counteracting CPA toxicity and its broader implications for health and food safety. Extra virgin olive oil (EVOO), an important component of the Mediterranean diet, is recognized for its health-promoting properties, particularly in preventing chronic diseases [10]. These benefits are largely attributed to its high content of monounsaturated fatty acids (MUFAs) and bioactive compounds, such as polyphenols. MUFAs, due to their resistance to oxidation, contribute to oxidative stability, while phenolic compounds play a key role in the antioxidant and anti-inflammatory properties of EVOO [11,12]. These compounds comprise several chemical families, including secoiridoids (oleuropein and ligstroside derivatives), phenolic alcohols such as hydroxytyrosol and tyrosol, as well as flavonoids, lignans and phenolic acids. Among these, secoiridoids are typically the most abundant and biologically relevant constituents in EVOO. Recent studies using LC–MS have identified a highly complex phenolic profile in Spanish EVOOs, with more than 40 compounds detected and a predominance of oleuropein and ligstroside aglycones, which have been associated with antioxidant and neuroprotective properties [13].

OLE, one of the main phenolic compounds in olive oil, undergoes hydrolysis to form HT and has been shown to exhibit neuroprotective properties by modulating apoptosis, autophagy, and inflammatory pathways. These effects are associated with a reduced risk of developing Alzheimer’s disease and other neurological disorders [14]. Additionally, the literature indicates that OLE reduces oxidative tissue damage by scavenging free radicals, further supporting its potential as a therapeutic agent in combating oxidative stress-related neurological conditions [15]. TYR, another important phenolic compound in EVOO, has demonstrated therapeutic potential for various chronic conditions, particularly through its ability to combat oxidative stress [16]. In diabetes, it protects pancreatic β-cells by modulating JNK signaling and alleviating endoplasmic reticulum stress, thereby preventing apoptosis. TYR also exhibits anti-inflammatory properties by inhibiting pro-inflammatory mediators through NF-κB inactivation and shows hepatoprotective effects by modulating oxidative stress, lipid metabolism and intracellular signaling [17]. Additionally, TYR has been associated with protection against neurological disorders by counteracting neuroinflammation and oxidative damage, making it a promising candidate for studies involving neuronal cell models such as SH-SY5Y cells [18].

The objectives of this study were: (i) to investigate the oxidative stress induced by CPA in SH-SY5Y cells; (ii) to evaluate the effects of TYR and OLE on CPA-induced cytotoxicity in SH-SY5Y cells; and (iii) to determine the antioxidant effect of EVOO extract in SH-SY5Y cells after CPA exposure.

## 2. Results

### 2.1. Measurement of Intracellular Reactive Oxygen Species

**Figure 1:**
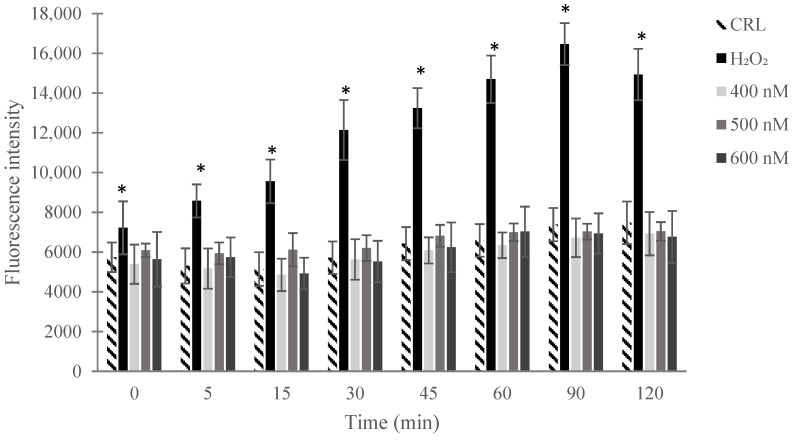
Intracellular reactive oxygen species (ROS) production in SH-SY5Y cells treated with 400, 500 and 600 nM cyclopiazonic acid (CPA) at 0, 5, 15, 30, 45, 60, 90 and 120 min. Data are expressed as mean ± SEM of three independent experiments (*n* = 3). (*) *p* ≤ 0.05 indicates a significant difference compared with time-matched untreated control cells (CRL). H_2_O_2_ was used as a positive control.

The early intracellular generation of ROS in SH-SY5Y cells exposed to CPA was analyzed using a dichlorofluorescein diacetate (DCFH-DA) probe. The cells were exposed to CPA (400, 500 and 600 nM), and increases in fluorescence were measured at intervals up to 2 h (0, 5, 15, 30, 45, 60, 90 and 120 min). No significant increase in ROS-induced fluorescence was observed over a 2 h period at any CPA concentration tested (Figure 1). The results confirmed the absence of oxidative stress produced by CPA.

### 2.2. Mitochondrial Membrane Potential

**Figure 2:**
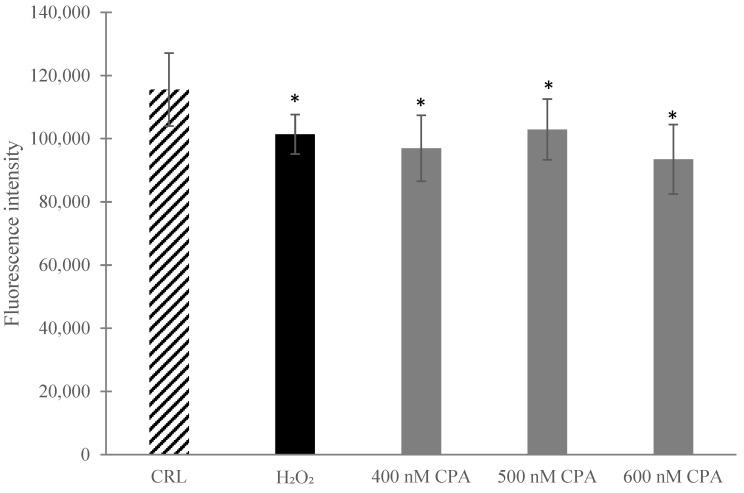
Mitochondrial membrane potential in SH-SY5Y cells exposed to cyclopiazonic acid (CPA) at 400, 500 and 600 nM for 24 h. The data are expressed as the mean ± SEM of three independent experiments (*n* = 3). (*) *p* ≤ 0.05 indicates significant difference with respect to the control (CRL). H_2_O_2_ was used as a positive control.

Changes in ΔΨm were assessed using Rh-123 dye, which reflects a shift in fluorescence as the dye moves from the mitochondria to the cytosol. As shown in Figure 2, the exposure of SH-SY5Y cells to increasing concentrations of CPA (400, 500 and 600 nM) resulted in a reduction in ΔΨm of 16%, 11% and 19%, respectively, compared to the control.

### 2.3. Measurement of Lipid Peroxidation

**Figure 3:**
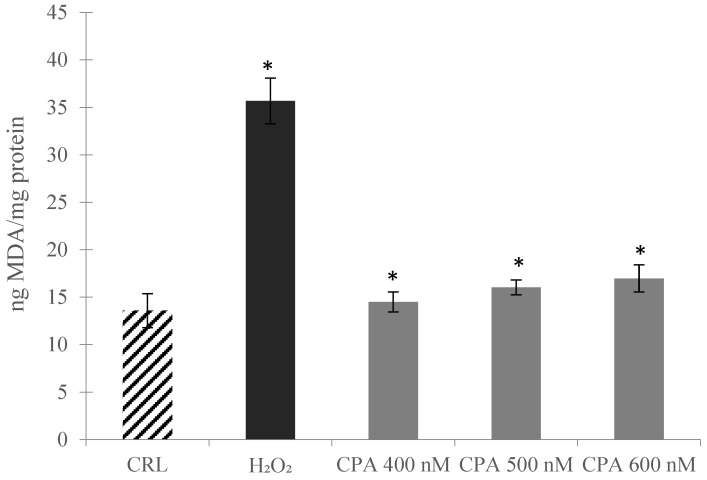
Determination of lipid peroxidation in SH-SY5Y cells exposed to cyclopiazonic acid (CPA) at 400, 500 and 600 nM for 24 h. Results are expressed as mean ± SEM of three independent experiments (*n* = 3) in ng MDA/mg protein measured by the Bradford method. (*) *p* ≤ 0.05 indicates a significant difference with respect to the control (CRL). H_2_O_2_ was used as a positive control.

The LPO was determined by the TBARS method, which measured the MDA-TBA adducts formed in SH-SY5Y cells after exposure to CPA (400, 500 and 600 nM) for 24 h. The results indicate that CPA induced a concentration-dependent increase in MDA production compared to the control, with increases of 7%, 18% and 25% at 400, 500 and 600 nM, respectively (Figure 3).

### 2.4. Measurement of Mitochondrial Superoxide (MitosoxRED)

**Figure 4:**
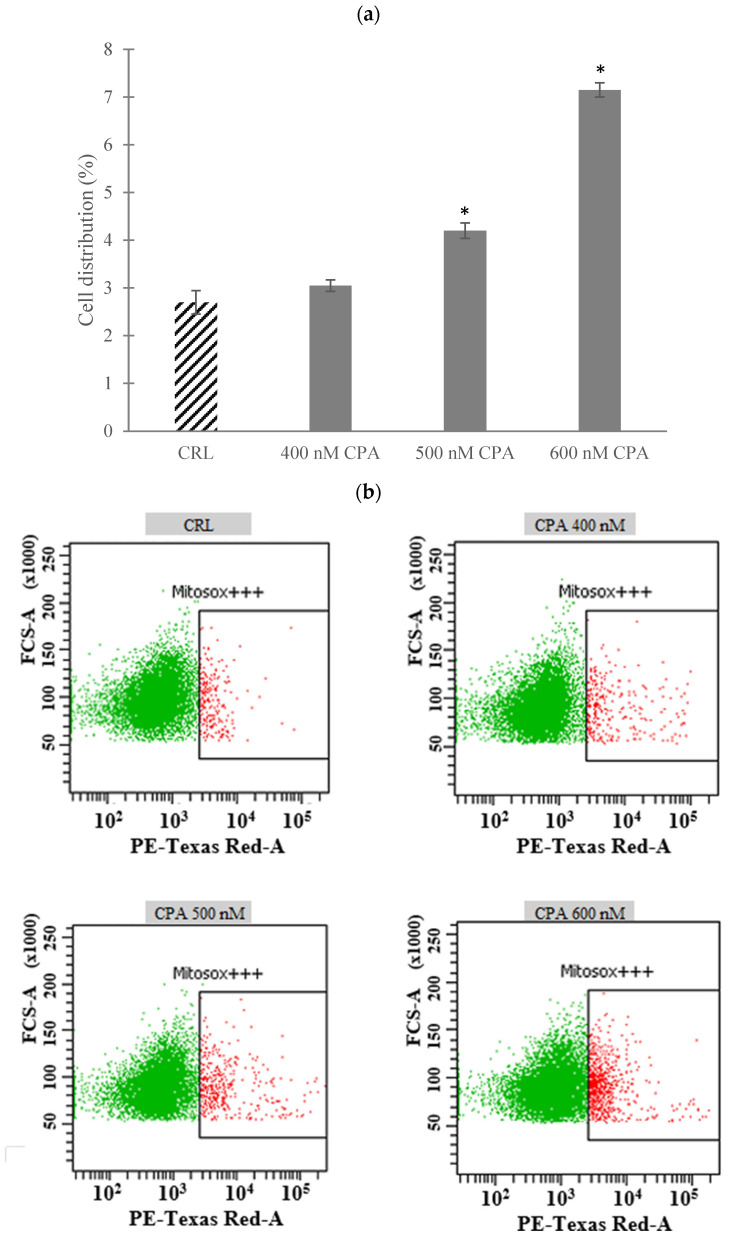
Mitochondrial superoxide generation in SH-SY5Y cells exposed to cyclopiazonic acid at 400, 500 and 600 nM for 24 h. Mitochondrial superoxide was measured by MitoSOX™ Red Mitochondrial Superoxide Indicator by flow cytometry. (**a**) Data are expressed as mean ± SEM of three independent experiments (*n* = 3). (*) *p* ≤ 0.05 indicates significant difference with respect to the control (CRL). (**b**) Dot plot of control cells (≤1% DMSO) and cells exposed to 400, 500 and 600 nM CPA for 24 h; red dots within the gated region (MitoSOX+++) represent cells with high mitochondrial oxidative stress levels, whereas green dots correspond to cells exhibiting basal or lower mitochondrial superoxide production.

The levels of mitochondrial superoxide were assessed in SH-SY5Y cells exposed to CPA (400, 500 and 600 nM) for 24 h using the mitochondria-targeted superoxide-sensitive fluorogenic probe MitoSOX™ Red. Our results indicated that CPA significantly increased mitochondrial superoxide production in SH-SY5Y in a concentration-dependent manner. While the lowest concentration of CPA (400 nM) showed no significant differences, the higher concentrations (500 nM and 600 nM CPA) increased superoxide production by 56% and 165%, respectively, compared to the control (Figure 4a). The corresponding dot plots (Figure 4b) illustrate these changes visually: control cells displayed a homogeneous population with low basal MitoSOX™ Red fluorescence, whereas cells treated with 500 nM and 600 nM CPA exhibited a progressive shift of the population into the high-fluorescence region (MitoSOX+++), reflecting increased mitochondrial superoxide levels. Cells treated with 400 nM CPA showed a distribution similar to the control, consistent with the quantitative data. These observations confirm that CPA induces mitochondrial oxidative stress in SH-SY5Y cells in a concentration-dependent manner.

### 2.5. Gene Expression Analysis

**Figure 5:**
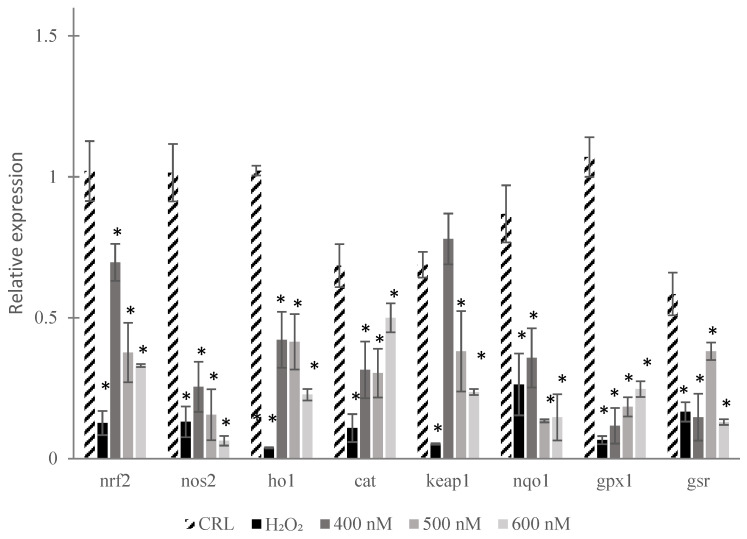
Analysis of the relative mRNA expression levels of nrf2, nos2, ho1, cat, keap1, nqo1, gpx1 and gsr quantified in SH-SY5Y cells after 24 h of CPA exposure using real-time PCR. The expression levels of the target genes were normalized against the corresponding 18S rRNA values and are presented as fold changes relative to the solvent-treated control. (*) *p* ≤ 0.05 indicates significant difference with respect to the control (CRL).

In order to confirm that CPA induced oxidative stress in the treated cells, the mRNA expression levels of key oxidative stress-related genes (nrf2, nos2, ho1, cat, keap1, nqo1, gpx1 and gsr) were assessed by real-time PCR after 24 h of exposure to increasing concentrations of CPA (400, 500, and 600 nM). As shown in Figure 5, all the analyzed genes were suppressed in expression compared with the control, with the majority exhibiting a concentration-dependent downregulation. In particular, nos2, nqo1, ho1 and gsr showed the strongest repression at 600 nM of CPA, with mRNA levels decreasing by 93%, 83%, 79% and 78%, respectively. In addition, nrf2 expression was also reduced at all tested concentrations of CPA, with a 68% decrease at 600 nM. Similarly, gpx1 and cat were consistently downregulated across all concentrations of CPA. In contrast, for keap1, no significant changes were observed at the lowest concentration (400 nM), but expression was markedly decreased at 500 and 600 nM, with reductions of 45% and 66%, respectively. These results are in full agreement with the increase in oxidative stress markers observed in previous assays. The exact percentage changes in gene expression are summarized in Table 1.

### 2.6. Effect of Oleuropein, Tyrosol and EVOO Extract on SH-SY5Y Cell Viability

#### 2.6.1. Tyrosol (TYR) and Oleuropein (OLE)

**Figure 6:**
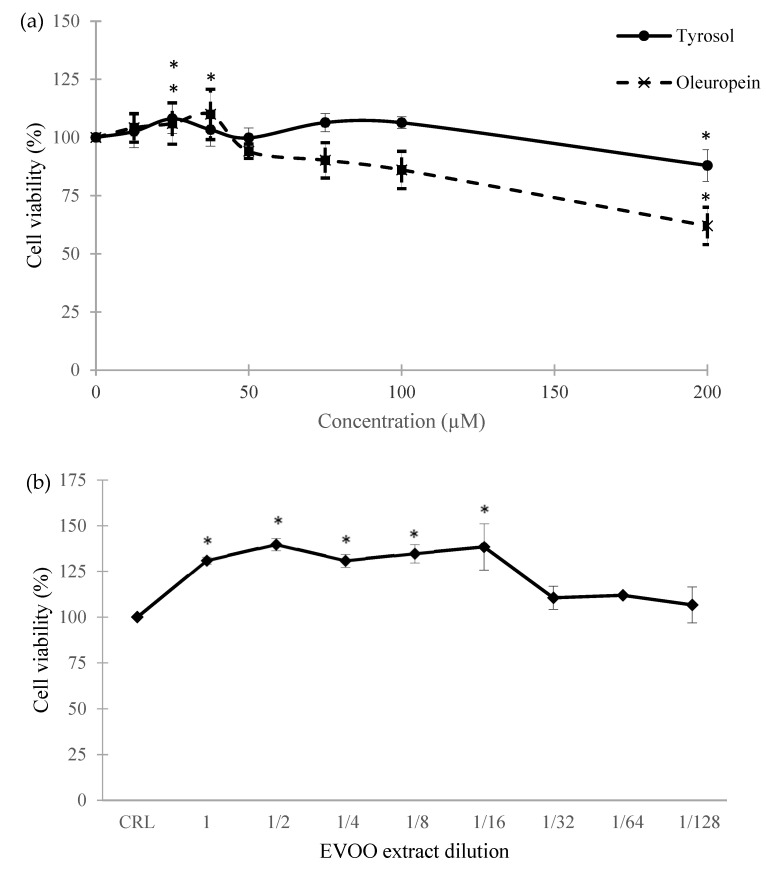
(**a**) Effect of oleuropein and tyrosol in SH-SY5Y cell viability after 24h of exposure; (**b**) effect of extra virgin olive oil (EVOO) extract (1) and its dilutions (1/2, 1/4, 1/6, 1/8, 1/16, 1/32, 1/64 and 1/128) in SH-SY5Y after exposure of 24 h. Data are expressed as mean ± SEM of three independent experiments (*n* = 3). (*) *p* ≤ 0.05 indicates significant difference with respect to the control (CRL).

SH-SY5Y cell viability after exposure to TYR and OLE for 24 h is shown in Figure 6a. Both compounds were non-cytotoxic at concentrations below 100 µM. At lower concentrations, both compounds increased cell viability: TYR at 25 µM increased cell viability by 8%, while OLE at 25 µM and 37.5 µM increased cell viability by 6% and 10%, respectively (Figure 6a).

The concentrations of TYR and OLE used in this study were selected based on previous findings. The TYR concentrations were chosen according to our prior work, where TYR demonstrated protective effects against mycotoxin-induced toxicity in Caco-2 cells [19]. Meanwhile, the concentrations for OLE were determined based on literature values [20]. Specifically, TYR and OLE concentrations in olive oil have been reported as 11.9 mg/kg and 11 mg/kg, respectively [21,22]. Additionally, considering that Spain’s average annual olive oil consumption per person is approximately 11.5 kg [23], the estimated daily intake of TYR and OLE is roughly 0.375 mg/day and 0.346 mg/day, respectively. This information allows for the estimation of daily intake equivalents corresponding to the tested concentrations of 25 µM for both TYR and OLE, providing a physiological context for the in vitro concentrations used. Although the estimated plasma concentrations of tyrosol (~0.5 µM) and oleuropein (~0.13 µM) following typical dietary intake are lower than the concentrations used in vitro, the selected 25 µM for both compounds was chosen to ensure detectable biological effects in cultured cells while remaining within a physiologically relevant order of magnitude. This approach is commonly used in cell-based studies of dietary polyphenols.

#### 2.6.2. Extra Virgin Olive Oil (EVOO) Extract

**Figure 7:**
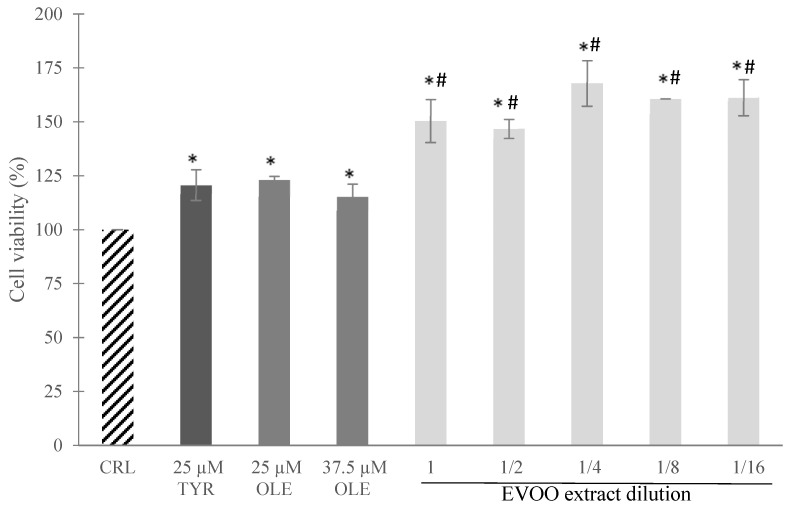
Effects of tyrosol (TYR, 25 µM), oleuropein (OLE, 25 and 37.5 µM) and extra virgin olive oil (EVOO) extract (1) and its dilutions (1, 1/2, 1/4, 1/8 and 1/16) in SH-SY5Y cell viability. Data are expressed as mean ± SEM of three independent experiments (*n* = 3). (*) *p* ≤ 0.05 indicates significant difference with respect to the control (CRL). (#) *p* ≤ 0.05 indicates significant difference with respect to TYR (25 µM) and OLE (25 and 37.5 µM).

SH-SY5Y cell viability after exposure to EVOO extract for 24 h is shown in Figure 6b. Undiluted EVOO extract increased cell viability by 28% (Figure 6b). Dilutions of the extract also enhanced cell viability: 1/2, 38%; 1/4, 27%; 1/8, 31%; 1/16, 34%, with no significant differences among them. Compared to TYR and OLE, EVOO extract produced a significantly higher increase in SH-SY5Y cell viability (Figure 7), suggesting that additional components in olive oil may contribute to its protective effect.

### 2.7. Effect of Tyrosol, Oleuropein and EVOO Extract in SH-SY5Y Cells Exposed to CPA

**Figure 8:**
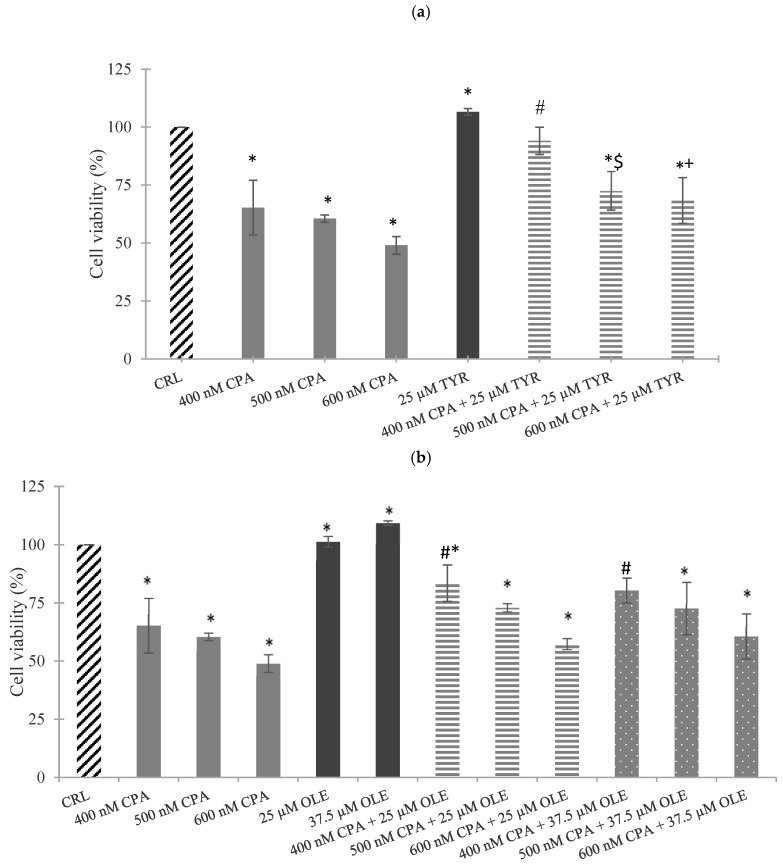
Protective effect of (**a**) tyrosol (TYR) and (**b**) oleuropein (OLE) in SH-SY5Y cells exposed to cyclopiazonic acid (CPA) at 400, 500 and 600 nM. Cells were simultaneously exposed to TYR (25 μM) or OLE (25 and 37.5 μM) and CPA for 24 h. Cell viability was determined using the MTT assay. (*) *p* ≤ 0.05 indicates significant difference with respect to the control (CRL). (#) *p* ≤ 0.05 indicates significant difference with respect to 400 nM CPA. ($) *p* ≤ 0.05 indicates significant difference with respect to 500 nM CPA. (+) *p* ≤ 0.05 indicates significant difference with respect to 600 nM CPA.

To evaluate whether the main EVOO phenolics and EVOO extract attenuate the cytotoxic effects of CPA in SH-SY5Y cells, the cells were simultaneously exposed to CPA (400, 500 and 600 nM) and TYR (25 μM) or OLE (25 and 37.5 μM). Cell viability under these combined treatments was determined using an MTT assay after 24 h of exposure (Figure 8). The combination of TYR (25 μM) and CPA at all concentrations assayed (400, 500 and 600 nM) significantly increased cell viability compared to the corresponding CPA controls by 45%, 20%, and 39%, respectively (Figure 8a). In contrast, 25 μM and 37.5 μM OLE significantly showed protective effect when combined with CPA at 400 nM only. Cell viability increased by 28% and 23%, respectively, compared to the CPA control. However, OLE in combination with 500 and 600 nM CPA did not show any cytoprotective effect (Figure 8b).

**Figure 9:**
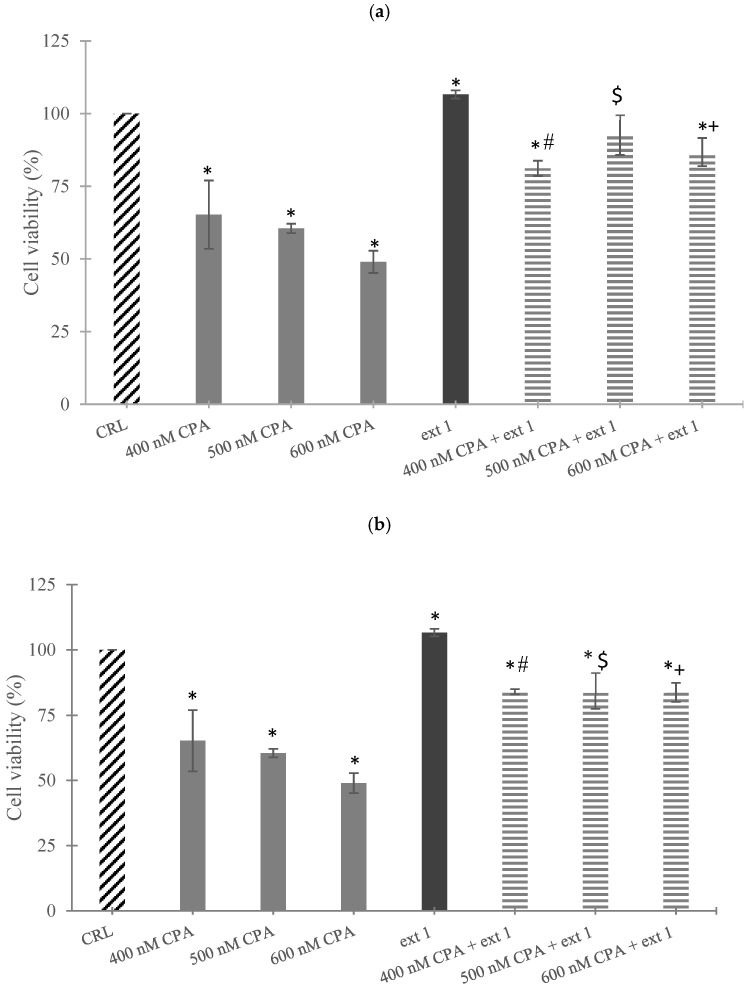
Effect of extra virgin olive oil (EVOO) extract on SH-SY5Y cell viability exposed to cyclopiazonic acid (CPA) at 400, 500 and 600 nM. Cells were simultaneously exposed to undiluted EVOO extract and CPA for 24 h. Cell viability was assessed using the (**a**) MTT assay and (**b**) PC method. (*) *p* ≤ 0.05 indicates significant difference with respect to the control (CRL). (#) *p* ≤ 0.05 indicates significant difference with respect to 400 nM CPA. ($) *p* ≤ 0.05 indicates significant difference with respect to 500 nM CPA. (+) *p* ≤ 0.05 indicates significant difference with respect to 600 nM CPA.

Figure 9 shows the effect of EVOO extract in SH-SY5Y cell viability, using MTT and PC assays after 24 h of exposure. The results obtained by both methods were consistent, showing no differences between them. Both methods indicated that the undiluted EVOO extract had a protective effect at all tested concentrations of CPA. Specifically, cell proliferation increased by 25%, 52% and 78%, respectively, compared to controls, by the MTT assay (Figure 9a), and by 29%, 38% and 71%, respectively, by the PC method (Figure 9b).

## 3. Discussion

The dichlorofluorescein assay revealed no significant early intracellular ROS generation after CPA exposure in SH-SY5Y cells. This suggests that CPA does not directly induce oxidative stress in SH-SY5Y cells under the assayed conditions. While H_2_O_2_ was used as a positive control to validate the DCFH-DA assay, it is important to note that DCFH-DA primarily detects cytosolic H_2_O_2_ and hydroxyl radicals and is relatively insensitive to mitochondrial superoxide. Consequently, it is not surprising that CPA did not induce a significant increase in DCF fluorescence in the range 0–120 min, whereas MitoSOX specifically detected mitochondrial ROS. These findings suggest that CPA primarily triggers oxidative stress within mitochondria rather than in the cytosol. Furthermore, it is possible that cytosolic ROS were generated outside the time window tested, representing a limitation of the experimental design that should be considered when interpreting the results. This finding aligns with results reported by Zingales et al. [24], where no significant intracellular ROS production was observed in SH-SY5Y cells exposed to sterigmatocystin (STE), a mycotoxin also produced by the Aspergillus genera, at concentrations between 0.78 and 3.12 μM. Despite this, both CPA and STE significantly increased mitochondrial superoxide production, highlighting the mitochondria as a critical target of these mycotoxins and suggesting a shared mechanism of mitochondrial oxidative stress. Specifically, in our study, CPA induced a concentration-dependent increase in mitochondrial superoxide levels, with increases up to 165%. Similarly, Zingales et al. [24] reported a significant induction of mitochondrial superoxide in SH-SY5Y cells exposed to STE. These results are of great value, as mitochondrial dysfunction is identified in many common pathologies, including cardiovascular diseases, neurodegeneration, metabolic syndrome, and cancer, underscoring the potential relevance of mitochondrial damage in the context of toxin exposure [25]. Similar results of those obtained in our study were observed by Tsai et al. [26]. Those authors reported that the rat cardiomyoblast cell line H9c2(2-1) was exposed to citrinin (CTN), a mycotoxin produced by Aspergillus and Penicillium species, at concentrations ranging from 25 to 75 μM. Mitochondrial superoxide levels were assessed using MitoSOX Red fluorescence, and flow cytometry analysis revealed a significant increase in the percentage of MitoSOX+ cells. These findings further support the role of mitochondrial oxidative stress as a critical mechanism underlying CTN-induced toxicity.

In addition to mitochondrial superoxide production, lipid peroxidation is another key hallmark of mycotoxin-induced oxidative stress. In our study, exposure to CPA in SH-SY5Y cells resulted in concentration-dependent increases in malondialdehyde (MDA) (the final product of polyunsaturated fatty acid oxidation), with increases of 7%, 18%, and 25% at CPA concentrations of 400, 500 and 600 nM, respectively. Similarly, Zingales et al. [24] reported significant increases in MDA levels in SH-SY5Y cells treated with STE, starting at concentrations as low as 0.78 μM. In line with these findings, Stockmann-Juvala et al. [27] observed elevated MDA levels in SH-SY5Y cells exposed to fumonisin B1 (FB1), an important Fusarium mycotoxin, highlighting that mitochondrial-derived ROS may drive lipid peroxidation across different mycotoxins. On the other hand, in vivo, Nogaim et al. [28] reported significant increases in MDA levels in the kidneys (36%), liver (57%) and brain (50%) of Wistar rats treated with ochratoxin A (OTA) at 10 mg/kg bw (LD_50_). OTA is a mycotoxin produced by Aspergillus and Penicillium species, and it is considered similar to CPA. Hence, these results underscore LPO as a hallmark of mycotoxin-induced damage in neuronal cells, likely driven by mitochondrial oxidative stress and membrane destabilization.

Moreover, CPA exposure led to concentration-dependent reductions in mitochondrial membrane potential (ΔΨm), with a decrease of up to 19%, indicating mitochondrial dysfunction that could impair ATP synthesis and energy metabolism. Similarly, Chen and Chan [29] observed a significant loss (approximately 50%) of mitochondrial membrane potential in HepG2 cells treated with 30 μM CTN for 24 h, suggesting that CPA and CTN share a mechanism of mitochondrial impairment affecting ΔΨm. In contrast, other authors reported no significant alterations in ΔΨm in SH-SY5Y cells exposed to 3.12 μM STE, suggesting that STE-induced cytotoxicity through other mechanisms, which does not involve mitochondrial membrane potential [24]. This comparison illustrates that while CPA, STE and CTN share some mitochondrial oxidative stress pathways, differences in ΔΨm response highlight toxin-specific mechanisms.

To further investigate the oxidative stress response triggered by CPA, the expression levels of eight key oxidative stress-related genes (nrf2, nos2, ho1, cat, keap1, nqo1, gpx1 and gsr) were assessed by qPCR following 24 h of exposure to increasing concentrations of CPA (400, 500 and 600 nM). The results revealed a clear downregulation of the majority of these genes in a concentration-dependent manner, suggesting that CPA impairs the antioxidant defense system, likely by inhibiting the nrf2 pathway rather than activating compensatory antioxidant responses. Particularly, nrf2, the most important transcription factor regulating the expression of enzymatic antioxidant response genes [30], was significantly repressed at all concentrations tested, with a reduction of over 65% at 600 nM. This was accompanied by marked downregulation of ho1 and nqo1, both canonical nrf2 target genes [31], indicating a compromised cytoprotective response. Similarly, nos2 expression was drastically reduced, suggesting impaired nitric oxide-mediated redox signaling. Interestingly, while keap1, the main negative regulator of nrf2 [32], did not show reduced expression at 400 nM, its levels declined substantially at higher concentrations. The simultaneous suppression of both nrf2 and keap1 is unusual, as keap1 downregulation would typically relieve inhibition on nrf2. This pattern may reflect global transcriptional suppression or cellular dysfunction induced by CPA, rather than a specific modulation of the nrf2–keap1 pathway. On the other hand, cat, gpx1 and gsr, enzymes directly involved in hydrogen peroxide detoxification and glutathione metabolism [33,34], were consistently downregulated across all concentrations of CPA, further supporting the notion of compromised antioxidant defenses. Similarly, Xue et al. [35] reported in an in vivo study using Gibel carp (*Carassius auratus gibelio*) that exposure to aflatoxin B1 (AFB1) induced oxidative stress, evidenced by increased levels of ROS and MDA, along with a significant reduction in the activity of key antioxidant enzymes. Furthermore, the expression of nrf2, a critical regulator of antioxidant defense, was downregulated in the AFB1-exposed group. In this study, the simultaneous downregulation of all three genes (nrf2, ho1 and nqo1) may be indicative of an inhibition of the nrf2 signaling pathway. It is evidently that in such a state, the cell is no longer capable of activating its defense response after exposure to CPA. Consequently, it can be hypothesized that CPA diminishes the antioxidant capacity of the cell and engenders heightened vulnerability to oxidative stress, inflammation and DNA damage, potentially leading to progression of cell damage, accelerated ageing or oxidative stress-associated diseases. In addition to nrf2 being downregulated, keap1 is also downregulated, which may indicate that the nrf2–keap1 signaling pathway is suppressed in general, either by direct repression of CPA or by transcriptional dysregulation resulting from cellular dysfunction, chronic stress or inflammation resulting from CPA exposure. Conversely, nos2 downregulation has been demonstrated to result in a diminution of the inducible inflammatory response. As demonstrated in the extant literature, the downregulation of cat, gpx and gsr has been shown to have a significant impact on the cell’s capacity to inactivate reactive oxygen species. Oxidative stress, a condition that has been demonstrated to compromise vital cellular structures and contribute to the development or progression of numerous chronic and degenerative diseases, is further exacerbated by CPA. In conclusion, the findings from this study, together with previous assays, indicate that exposure to CPA increases the vulnerability of SH-SY5Y cells to oxidative stress, elevating the risk of cell damage and inflammation. This effect is likely mediated through the disruption of mitochondrial function and concurrent suppression of key antioxidant defense genes.

The protective potential effect of natural compounds against CPA-induced toxicity was evaluated using EVOO extract and its main individual phenolic components, TYR and OLE. EVOO is known for its rich phenolic content and antioxidant properties, making it a relevant candidate for mitigating toxin-induced damage in neuronal cells. Previous studies have demonstrated that EVOO and its phenolic compounds exhibit neuroprotective effects, including reducing oxidative stress, enhancing blood–brain barrier (BBB) integrity and modulating neuroinflammation—key mechanisms involved in neurodegenerative diseases such as Alzheimer’s disease [36,37,38]. These EVOO properties could also play a role in protecting neuronal cells from CPA-induced toxicity, supporting its potential for mitigating neurodegeneration. Our findings highlight both the potential cytoprotective effect of EVOO extract and the variable responses of its individual phenolics in alleviating the toxic effects produced by CPA. EVOO extract showed cytoprotective effects across all tested dilutions. Cell viability increased by approximately 30% at dilutions of 1/2 to 1/16, as well as with the undiluted extract. These results align with those reported by Chiesi et al. [20], where EVOO extract enhanced Caco-2 cell viability, with the greatest effect (36%) observed at intermediate dilutions (1/2, 1/4 and 1/6). Both studies emphasize the role of EVOO in alleviating mycotoxin-induced cytotoxicity across mycotoxins with different chemical structures. For instance, Chiesi et al. [20] demonstrated that EVOO reduced the toxic effects of 25–100 µM alternariol (AOH) in Caco-2 cells. Similarly, our study shows that EVOO extract provided potential protection against CPA-induced damage at all tested toxin concentrations. The enhanced efficacy of EVOO extract may be explained by its complex phenolic composition. EVOO contains a wide range of bioactive compounds, including secoiridoids (oleuropein and ligstroside derivatives), and phenolic alcohols such as hydroxytyrosol and tyrosol, as well as flavonoids, lignans and phenolic acids, which have been widely associated with antioxidant and neuroprotective activities. In this context, López-Bascón et al. [13] characterized EVOOs from Granada (southern Spain), identifying more than 40 phenolic compounds by LC–MS, with a predominance of secoiridoids—particularly oleuropein and ligstroside aglycones—as well as relevant amounts of hydroxytyrosol and tyrosol, which were strongly associated with neuroprotective properties. Although the EVOO used in the present study was obtained from a local commercial source in Spain and was not individually characterized, it is reasonable to assume a comparable phenolic profile, as Spanish EVOOs from Mediterranean regions typically share a similar composition. In contrast, the effects of TYR and OLE were less consistent and were dependent on their concentration. Chiesi et al. [20] observed that 50 µM OLE provided a slight protective effect against AOH-induced damage, but higher concentrations exacerbated cytotoxicity. Specifically, higher doses of OLE (100 µM) reduced cell viability by 20% with 12.5 µM AOH. Our results showed similar concentration-dependent effects. When 25 µM OLE was combined with 400 nM CPA, it increased cell viability by 28%, suggesting some protective effect. Previously, it was demonstrated that OLE exhibits potent cytoprotective effects against hydrogen peroxide-induced oxidative damage in human trophoblast cells (HTR-8/SVneo). It significantly enhances antioxidant status, prevents protein and lipid damage, and reduces iNOS levels while also lowering the expression of pro-inflammatory cytokines IL-6 and TNF-α. Moreover, OLE preincubation and H_2_O_2_ exposure decreased levels of MDA and lactate dehydrogenase (LDH) activity, indicating its ability to mitigate LPO in trophoblast cells [39]. These findings underscore OLE’s protective role in attenuating oxidative damage and restoring antioxidant functionality across different cell models. Similarly, this effect was also observed in human embryonic kidney (HEK-293) cells, where OLE treatment markedly reduced LPO, evidenced by lower MDA production, and prevented H_2_O_2_-induced apoptosis, as reported by Maalej et al. [40]. In contrast, Chiesi et al. [20] found that 50 µM TYR effectively prevented AOH-induced cytotoxicity, increasing Caco-2 cell viability significantly. In our study, 25 µM TYR consistently enhanced cell viability when combined with CPA, with significant increases of up to 45%. This suggests that TYR offers greater efficacy in cytoprotection compared to OLE. Furthermore, in previous work, we demonstrated that a low concentration of TYR (25 µM) was able to attenuate the toxic effects induced by a low exposure to T-2 toxin (15 nM) in Caco-2 cells, further supporting the protective role of tyrosol in reducing cell damage caused by different mycotoxins [19]. Similarly, Schaffer et al. [41] reported that low amounts of hydroxytyrosol prevented oxidative stress in PC12 cells. Thus, our findings align with results obtained in the literature, reinforcing that TYR may have a stronger and more consistent protective effect compared to OLE under our experimental conditions. These findings suggest that the combined interactions within the complex matrix of EVOO extract enhance its cytoprotective effects as opposed to the isolated activity of individual phenolics. Previous studies have highlighted the importance of such interactions in modulating oxidative defense and preserving mitochondrial integrity [42,43,44]. Our results support this possibility, demonstrating the cytoprotective effect of EVOO extract compared to its isolated components.

## 4. Conclusions

This study highlights the neurotoxic effects of CPA in SH-SY5Y cells and demonstrates the cytoprotective potential of EVOO extract and its main phenolic components, TYR and OLE. CPA exposure did not induce early intracellular ROS but significantly increased mitochondrial superoxide levels, lipid peroxidation and disrupted mitochondrial membrane potential, indicating that mitochondrial oxidative stress is a central mechanism of its cytotoxicity. Gene expression analysis further revealed suppression of the nrf2–keap1 pathway and the antioxidant defense system, reinforcing the role of oxidative imbalance in CPA-induced toxicity. Treatment with EVOO extract enhanced cell viability across all tested CPA concentrations, supporting its effectiveness in mitigating mitochondrial oxidative damage. These findings provide mechanistic insights into how EVOO phenolics can modulate cellular oxidative stress and suggest their potential as protective agents in neurotoxicological contexts where mitochondrial dysfunction and oxidative stress are key contributors to cell injury. Future studies should evaluate EVOO’s protective effects against mycotoxin exposure in in vivo models to assess its potential clinical relevance.

## 5. Materials and Methods

### 5.1. Reagents

The reagent-grade chemicals and cell culture components used, namely, Dulbecco’s Modified Eagle’s Medium-F12 (DMEM/F-12), penicillin, streptomycin, trypsin/EDTA solutions, fungizone, phosphate buffered saline (PBS), Hank’s Balanced Salt Solution (HBSS), Fetal Bovine Serum (FBS), methylthiazoltetrazolium salt (MTT) dye, thiobarbituric acid (TBA), deferoxamine mesylate salt (DFA), di-ter-butylmethylphenol (BHT), 1,1,3,3-tetramethoxipropane (TMP), 2′,7′dichlorodihydrofluorescein diacetate (H_2_-DCFDA), 4′,6-diamidine-2′-phenylindole dihydrochloride (DAPI), H_2_O_2_, glacial acetic acid, rhodamine 123 (Rh-123), Coomassie Brilliant Blue, sodium hydroxide (NaOH), dimethyl sulfoxide (DMSO) and Sorensen’s glycine buffer were acquired from Sigma-Aldrich (Burlington, MA, USA). MitoSOX^TM^ Red Mitochondrial Superoxide Indicator was acquired from Thermo Fisher Scientific (Waltham, MA, USA). Methanol (MeOH) and NaCl were purchased from Merck Life Science S.L. (Burlington, MA, USA) and the bovine serum albumin (BSA) was purchased from Rockland Inc. (Bedford, PA, USA). Deionized water (resistivity <18 MΩ cm) was obtained using a Milli-Q water purification system (Millipore, Bedford, MA, USA). Forward and reverse primers for the genes nrf2, nos2, ho1, cat, keap1, nqo1, gpx1 and gsr were obtained from Invitrogen (Carlsbad, CA, USA).

Standards of CPA (≥98% purity), TYR (≥98% purity) and OLE (≥98% purity) were acquired from Sigma-Aldrich (Barcelona, Spain). EVOO was acquired from a local supermarket located in Valencia (Spain). Stock solutions of CPA, TYR and OLE were prepared in DMSO at appropriate working concentrations and maintained at −20 °C in the dark. Final concentrations of CPA, TYR and OLE in the assay were attained by adding the culture medium. The final DMSO concentration in the culture medium was ≤1% (*v*/*v*). Control cells were exposed to the same concentration of DMSO.

### 5.2. Cell Culture

SH-SY5Y human neuroblastoma cells were procured from the American Type Culture Collection (ATCC CRL-2266). These cells were cultured in a monolayer in DMEM/F-12, supplemented with 10% FBS, 0.2% fungizone, 100 U/mL penicillin and 100 mg/mL streptomycin. The incubation conditions were pH 7.4, 5% CO_2_ at 37 °C and 95% air atmosphere at constant humidity. The cells were subcultured approximately twice weekly, limited to fewer than 20 sub-passages. This approach ensured genetic stability, preventing phenotypic changes that might influence responses to treatments or experimental conditions, thus safeguarding the accuracy and reproducibility of the results. SH-SY5Y cells were subcultured following trypsinization, using a 1:2 split ratio. The medium was changed every 2–3 days. Mycoplasma absence was regularly verified through a MycoAlert™ PLUS Mycoplasma Kit (Lonza, Rockland, ME, USA). SH-SY5Y cells were provided by the Central Service for Experimental Research (SCSIE) of the University of Valencia (Valencia, Spain).

### 5.3. Intracellular ROS Generation

Intracellular ROS production was assessed in SH-SY5Y cells by adding H_2_-DCFDA [45]. The H_2_-DCFDA is taken up by cells and then deacetylated by intracellular esterases; the resulting non-fluorescent 2′,7′-dichlorodihydrofluorescein (H_2_-DCF) is converted to greatly fluorescent dichlorofluorescein (DCF) when oxidized by ROS. In brief, 4 × 10^4^ cells/well were seeded in a 96-well black polystyrene culture microplate. Once cells exhibited 90% confluence, the culture medium was replaced, and cells were loaded with 20 µM H2-DCFDA in a fresh medium for 20 min in the dark. Subsequently, the H2-DCFDA was removed and replaced by CPA (400, 500 and 600 nM) and the fluorescence emitted by the DCF was monitored at different times (0, 5, 15, 30, 45, 60, 90 and 120 min) on a multimode microplate reader (Biotek Synergy H1; Agilent, Kennesaw, GA, USA), at excitation/emission wavelengths of 485/535 nm, respectively. Determinations were performed in three independent experiments with three replicates each and the results were expressed as increase (%) in fluorescence in respect to control cells. Hydrogen peroxide (H_2_O_2_) (500 µM) was used as a positive control. Early time points (up to 2 h) were selected for ROS measurement because ROS generation is a rapid and transient cellular response that precedes downstream effects such as changes in cell viability or apoptosis, which were assessed after 24 h. This approach allows capturing the initial oxidative response induced by CPA.

CPA concentrations used in this study (400, 500 and 600 nM) were selected based on previous findings conducted in our laboratory in SH-SY5Y cells. In our last study, the mean inhibitory concentration (IC50) of CPA in SH-SY5Y after 24 h was determined to be 864 nM [46]. Therefore, it was appropriate to select concentrations lower than the IC_50_ in this work. Consequently, 400, 500 and 600 nM of CPA were selected.

### 5.4. Measurement of Mitochondrial Membrane Potential (ΔΨm)

The mitochondrial membrane potential (ΔΨm) in living cells was assessed using Rh-123, following the method by Mallebrera et al. [47]. Rh-123 is a green-fluorescent dye that can be absorbed by living cellular mitochondria. In brief, SH-Y5Y cells were seeded in 96-well black culture microplates at a density of 4 × 10^4^ cells/well. After 24 h of CPA exposure (400, 500 and 600 nM), the medium was removed and the cells were incubated in darkness with 5 μM Rh-123 in fresh medium for 15 min at 37 °C. The fluorescence was measured using a multimode microplate reader (Biotek Synergy H1; Agilent, Kennesaw, GA, USA) at excitation/emission wavelengths of 485/535 nm, respectively. Results are expressed as decrease in fluorescence compared to the control (1% DMSO). Three independent experiments were performed. H_2_O_2_ (500 µM) was used as a positive control.

### 5.5. Lipid Peroxidation Assay

Lipid peroxidation (LPO) assay was conducted by assessing the formation of reactive thiobarbituric acid reactive substances (TBARS), according to the procedure reported by Ferrer et al. [45]. TBARS allows the determination of the production of a red adduct between thiobarbituric acid (TBA) and malondialdehyde (TBAR). MDA is the end product of polyunsaturated fatty acid oxidation, which can react with TBA to form an adduct. This adduct can be quantified colorimetrically through spectrometric analysis and serves as a biomarker to confirm that the LPO process has occurred. Briefly, 7 × 10^5^ cells/well were seeded in six-well plates. Once the cells reached 90% confluence, cells were exposed to CPA (400, 500 and 600 nM) for 24 h. Then, the medium was removed and cells were homogenized in 150 mM sodium phosphate buffer (NaH_2_PO_4_) pH 7.4 and lysate with a polytron (Ultra-Turrax T8 IKA^®^-WERKE). At once, cells were heated for 20 min at 100 °C in water bath under acidic condition in the presence of 0.5% TBA, 1.5 mM DFA and 3.75% BHT. Afterwards, the samples were cooled on ice for 5 min and then centrifuged at 4000 rpm for 15 min. The absorbance was measured at 532 nm. Three independent experiments were performed. Results were expressed as ng of MDA/mg of protein measured by the Bradford method. H_2_O_2_ (500 µM) was used as a positive control.

### 5.6. Mitochondrial Superoxide Measurement

Mitochondrial superoxide levels were assessed using MitoSOX™ Red Mitochondrial Superoxide Indicator (M36008, Thermo Fisher Scientific), a fluorogenic dye specifically designed for the detection of superoxide in live cell mitochondria. When present in the mitochondria, MitoSOX™ Red is selectively oxidized by superoxide rather than by other ROS. In its oxidized form, the probe shows excitation/emission maxima at 510/580 nm, respectively. The assay was performed as described by Zingales et al. [24] with some modifications. In brief, 7 × 10^5^ cells/well were seeded in six-well plates. After 24 h of CPA exposure (400, 500 and 600 nM), SH-SY5Y cells were trypsinized and MitoSOX Red reagent was added at a final concentration of 5 μM in HBSS buffer to the cells. After incubation for 15 min at 37 °C in the dark, the medium was removed and the cells were washed three times with pre-warmed HBSS buffer. An amount of 10,000 live cells was acquired and analyzed by a BD LSRFortessa (BD Biosciences, San Jose, CA, USA) using flow cytometry. Dead cells, stained with DAPI (20 μg/μL), were not considered. Three independent experiments were carried out. Results were expressed as the percentage of the cell population marked as MitoSOX+++ by flow cytometry.

### 5.7. RNA Extraction and Quantification

For the evaluation of oxidative stress gene expression, cells were seeded at a density of 7 × 10^5^ cells/well in a 6-well plate, treated with different concentrations of CPA (400, 500 and 600 nM). Then, total RNA was extracted from SH-SY5Y cells using a ReliaPrep™ RNA Cell Miniprep System kit. The RNA obtained from each sample was initially evaluated for both quantity and quality with a Nanodrop2000 spectrophotometer (Thermo Scientific). Measured concentrations ranged from 200 to 700 ng/μL, with 260/280 and 260/230 absorbance ratios equal to or greater than 2.0, confirming high purity. The RNA samples were kept at −20 °C until being diluted to a final concentration of 100 ng/μL in Milli-Q grade water for subsequent reverse transcription into complementary DNA (cDNA).

### 5.8. Reverse Transcription and qPCR Reaction

First, cDNA synthesis was carried out using 5 μL of total RNA (500 ng), following the protocol outlined in the TaqMan™ MicroRNA Reverse Transcription Kit (Thermo Fisher Scientific, Madrid, Spain). Quantitative PCR (qPCR) was performed for all primer pairs, and a single amplification product was confirmed for each gene through melting curve analysis. The amplification efficiency of each primer was assessed using a standard curve generated by serial dilutions of cDNA (dilution factor = 2), with each dilution tested in triplicate. The gene-specific primers used in this study are listed in Table 2. Real-time amplification reactions were conducted in 96-well plates with SYBR Green detection reagent, using the StepOne Plus Real-Time PCR System (Applied Biosystems, Foster City, CA, USA)). Each reaction mixture was prepared in a total volume of 10 μL containing 3 μL of 1:2 diluted cDNA, 2 μL of the amplification primer mix (forward/reverse primers for each gene at 2.5 μM) and 5 μL of SYBR Green (Applied Biosystems, Foster City, CA, USA)). Each experiment was conducted in two independent replicates, with three technical replicates per experiment. The qPCR protocol consisted of a 5 min incubation at 95 °C followed by 40 cycles of 30 s at 95 °C and 30 s at 60 °C, 40 s at 72 °C and a final melt curve from 58 °C to 95 °C. Baseline corrections, threshold cycle (Ct) values and primer efficiency parameters were automatically calculated using StepOne Plus Software version 2.3 (Applied Biosystems). Non-template controls (NTCs) were prepared using water instead of cDNA. 18S rRNA was used as the reference gene because it is widely reported as one of the most stable and abundantly expressed housekeeping genes across multiple cell types and experimental conditions [48]. Studies have shown that 18S rRNA exhibits minimal variability and is therefore suitable for normalization in qPCR experiments, particularly when targeting mRNA transcripts with moderate to high expression levels. Its use is consistent with common practice in the field for this type of sample and experimental design [49].

### 5.9. Preparation of an Extract of Extra Virgin Olive Oil

The EVOO sample was obtained from a local supermarket located in Valencia (Spain) and kept at room temperature until analysis. The EVOO extract was prepared according to Chiesi et al. [20] with some modifications: 10 g of sample was dissolved in 5 mL hexane, and the solution was extracted three times with 10 mL of a mixture of H_2_O:MeOH (60:40). The mixtures were centrifuged for 5 min at 3500 rpm. The obtained extract was brought to dryness in a vacuum rotary evaporator at 65 °C. The residue was dissolved in 2.5 mL DMEM and stored at −20 °C until it was used. MTT and Total Protein Content (PC) assays were performed to assess whether EVOO extract affects the viability of SH-SY5Y cells, as described later. For these assays, the following dilution series of the extract with medium was performed: 1, 1/2, 1/4, 1/6, 1/8, 1/32, 1/64 and 1/128. The EVOO extract and its dilutions were simultaneously exposed to CPA (400, 500 and 600 nM) in order to evaluate the cytoprotective effects of EVOO on SH-SY5Y cells exposed to CPA.

### 5.10. In Vitro Cytotoxicity

SH-SY5Y cells have been widely employed in in vitro toxicological studies to assess cell proliferation and survival. SH-SY5Y cells were cultured in 96-well tissue-culture plates by adding 200 μL/well at a density of 2 × 10^4^ cells/well. After the cells reached 80% confluence, the culture medium was replaced by fresh medium containing different concentrations of CPA (400, 500 and 600 nM), tyrosol (12.5–200 µM), oleuropein (12.5–200 µM) or EVOO extract. Then, plates were incubated in darkness at 37 °C, 5% CO_2_ and 95% air atmosphere at constant humidity for 24 h. Cell viability was determined by an MTT assay. The MTT assay is based on the capacity of viable cells to metabolize, via a mitochondrial-dependent reaction, the yellow tetrazolium salt to an insoluble purple formazan. MTT assay was carried out according to the procedure reported by Ruiz et al. [50]. In summary, the medium containing CPA was removed and each well received 200 μL of fresh medium containing 50 μL of MTT. The plates were returned to the incubator and kept in the dark for 3 h. Then, the MTT solution was removed and 200 μL of DMSO was added followed by 25 μL of Sorensen’s glycine buffer. The absorbance was measured at 595 nm using an automatic ELISA plate reader (MultiSkanEX, Thermo Scientific, Walthman, MA, USA).

The total protein content (PC) was also determined. The PC method relies on the increase in absorbance of Coomassie Brilliant Blue dye upon its interaction with proteins. The assay was conducted using the same 96-well plates previously used for the MTT test (CPA + EVOO extract), following the procedure reported by Taroncher et al. [51]. Briefly, the plates were washed with PBS, followed by the addition of 200 μL of 0.1 N NaOH to each well to dissolve the proteins. After 2 h of incubation, 170 μL of the NaOH solution was removed from each well, and 180 μL of a 22% diluted Coomassie Brilliant Blue solution was added. The plates were then left at room temperature for 30 min and the absorbance was measured at 595 nm using an automatic ELISA plate reader (MultiSkanEX, Thermo Scientific, Walthman, MA, USA).

For MTT and PC assays, the values of cell viability were expressed as a percentage of the solvent (≤1% DMSO). Each exposure time was tested three times in independent experiments (8 replicates per experiment). The IC_50_ values were determined using SigmaPlot version 11 software (Systat Software Inc., GmbH, Erkrath, Germany).

### 5.11. Statistical Analysis

Statistical analysis of data was carried out using Statgraphics version 19 (IBM Corp., Armonk, NY, USA). Data were expressed as the mean ± standard error of the mean (SEM) from three independent experiments. The Student’s *t*-test was also performed for paired samples. For multiple comparison, one-way analysis of variance (ANOVA) was applied followed by the Tukey’s HSD *post hoc* test. A *p*-value of *p* ≤ 0.05 was considered statistically significant.

## Figures and Tables

**Table 1:** Percentage changes in gene expression in SH-SY5Y cells after 24 h of CPA treatment. Positive values represent downregulation, while negative values indicate upregulation relative to the control (CRL).

Gene	CPA 400 nM	CPA 500 nM	CPA 600 nM
nrf2	31.70%	63.10%	67.60%
nos2	74.80%	84.60%	93.70%
ho1	58.80%	59.40%	77.80%
cat	54.00%	55.70%	27.00%
keap1	−13.2%	44.70%	65.70%
nqo1	58.90%	84.50%	83.10%
gpx1	89.10%	21.60%	76.90%
gsr	74.80%	30.20%	77.80%

**Table 2:** Gene-specific primers for qPCR experiments.

Gene Name	Gene Symbol	Optimum Temperature (°C)	Forward Primer	Reverse Primer
Nuclear factor erythroid 2–related factor 2	nrf2	64	CTGAACTCCTG	CGGTGGGTCT
			GACGGGACTA	CCGTAAATGG
Nitric oxide synthase 2	nos2	62	GGAGAAGGG	GCATTGGAAG
			GACGAACTCAGT	TGAAGCGTTTC
Heme oxygenase 1	ho1	64	AAGACTGCGTT	AAAGCCCTACA
			CCTGCTCAAC	GCAACTGTCG
18S rRNA	s18	62	CGGCTACCACA	GCTGGAATT
			TCCAAGGAA	ACCGCGGCT
Catalase	cat	62	CCAGAAGAA	GAGATCCGGACT
			AGCGGTCAAGAA	GCACAAAG
Superoxide dismutase 1	sod1	66	GGTGGGCCAAAGGATGAAGAG	CCACAAGCCA
				AACGACTTCC
Kelch-like ECH-associated protein 1	keap1	66	CGTCCTGCCA	GTGTCTTATCT
			ACTGTATCT	GGCTCGTAAC
NAD(P)H quinone dehydrogenase 1	Nqo1	64	CCCTGCGAA	CTTTCAGAATG
			CTTTCAGTATCC	GCAGGGACTC
Glutathione peroxidase 1	gpx	70	ATGAACGAGCT	CTAGGCACAGCT
			GCAGCGGCGC	GGGCCCTTG
Glutathione reductase	gsr	62	CCGGTGCCA	TTAGAACCCAG
			GCTTAGGAATA	GGCTGACAG

## Data Availability

The original contributions presented in this study are included in the article. Further inquiries can be directed to the corresponding author.
